# DDIT3 antagonizes innate immune response to promote bovine alphaherpesvirus 1 replication via the DDIT3-SQSTM1-STING pathway

**DOI:** 10.1080/21505594.2022.2044667

**Published:** 2022-03-08

**Authors:** Song Wang, Xiaomei Ma, Jin Guo, Fangxu Li, Tianhua Chen, Wenqing Ma, Chengqiang He, Hongmei Wang, Hongbin He

**Affiliations:** Ruminant Diseases Research Center, College of Life Sciences, Shandong Normal University, Jinan, China

**Keywords:** DDIT3, BoHV-1, STING, SQSTM1, innate immunity

## Abstract

DNA damage-inducible transcript 3 (DDIT3), a transcription factor, is typically involved in virus replication control. We are the first to report that DDIT3 promotes the replication of bovine viral diarrhea virus, an RNA virus, by inhibiting innate immunity. However, whether the DDIT3 gene participates in DNA virus replication by regulating innate immunity remains unclear. This study reported that DDIT3 suppressed the innate immune response caused by DNA viruses to promote bovine herpesvirus 1 (BoHV-1) replication. After BoHV-1 infection of Madin-Darby bovine kidney (MDBK) cells, upregulated expression of DDIT3 induced SQSTM1-mediated autophagy and promoted STING degradation. Overexpression of the SQSTM1 protein effectively reduced STING protein levels, whereas SQSTM1 knockdown increased STING protein levels. Coimmunoprecipitation experiments and confocal laser scanning microscopy revealed that the SQSTM1 protein interacts with and colocalizes with STING. Knockdown of SQSTM1 expression in DDIT3-overexpressing cell lines restored STING protein levels. Moreover, a dual-luciferase reporter assay revealed that DDIT3 directly binds to the bovine SQSTM1 promoter and induces SQSTM1 transcription. Overexpression of SQSTM1 promoted BoHV-1 replication by inhibiting IFN-β and IFN-stimulated genes (ISGs) production; silencing of SQSTM1 promoted the expression of IFN-β and ISGs to inhibit BoHV-1 replication. In conclusion, DDIT3 targets STING via SQSTM1-mediated autophagy to promote BoHV-1 replication. These results suggest a novel mechanism by which DDIT3 regulates DNA virus replication by targeting innate immunity. DDIT3 antagonizes the innate immune response to promote bovine alphaherpesvirus 1 replication via the DDIT3-SQSTM1-STING pathway.

## Introduction

DNA damage-inducible transcript 3 (DDIT3), also known as GADD153, CHOP and CHOP-10, is a 29 kD protein containing a transcription activation domain and a basic leucine zipper domain, the latter of which plays an essential role in the apoptosis induced by DDIT3 [1,2]. DDIT3 negatively regulates transcription by binding to CCAAT/enhancer binding protein family proteins to form heterodimers, and the protein also directly binds to DNA to initiate the synthesis of downstream target gene mRNA [3,4]. DDIT3 transcription and translation are induced by DNA damage, endoplasmic reticulum (ER) stress, hypoxia and starvation [5–7]. Misfolding of proteins during ER stress induced by viral infection leads to ER stress responses that might upregulate the expression of DDIT3. For example, coronavirus infectious bronchitis virus (IBV) and human astrovirus (HAstV) infection upregulate DDIT3 expression to promote virus release [8,9]. Hepatitis C virus envelope proteins and the SARS-CoV spike protein induce an unfolded protein response and trigger the PERK-eIF2α-ATF4 pathway, ultimately enhancing the expression of DDIT3 [10,11]. Recently, we reported that DDIT3 promotes the replication of bovine viral diarrhea virus (BVDV, an RNA virus) by suppressing the innate immune response [12], although it is not clear whether DDIT3 exerts a similar role in DNA virus infection.

During pathogen infection, induction of the innate immune response depends on the rapid and efficient recognition of pathogen-associated molecular patterns by host pattern recognition receptors [13]. cGAS is an important PRR that recognizes cytosolic DNA sensors and prompts IFN-I responses [14,15]. Upon DNA viral infection, cGAS binds to DNA virus cytosolic dsDNA, resulting in the production of the small second-messenger cGAMP in the presence of GTP and ATP [16,17]. The cGAMP generated interacts with and activates the adaptor protein stimulator of IFN genes (STING) at the endoplasmic reticulum membrane [16,18]. This binding of cGAMP to STING promotes the transport of STING from the endoplasmic reticulum membrane to the Golgi apparatus, where it recruits TBK1 to phosphorylate and activate IRF3, resulting in the activation of type I interferon signaling [19–21].

As the key downstream element in cGAS signaling, STING plays a critical role in innate immunity against DNA viruses [22,23]. However, a variety of DNA viruses have developed effective strategies to regulate the function of STING for survival. Human papilloma virus E7 and adenovirus E1A directly bind to STING to disrupt the cGAS-STING pathway [24]. In addition, hepatitis B virus polymerase directly interacts with STING and subsequently disrupts the K63-linked polyubiquitination of STING, thereby inhibiting the antiviral type I IFN response [25]. Herpesviruses use similar strategies to interfere with the cGAS-STING pathway, and many herpes simplex virus 1-encoded proteins (ICP27, VP24, UL41, UL24, UL36, VP11/12, VP22, UL37, γ134.5, UL46, and US3) have been reported to suppress the cGAS-STING-mediated antiviral pathway [26–37]. The murine cytomegalovirus M152 protein interacts with STING to antagonize the production of IFN-β [38], and Kaposi’s sarcoma-associated herpesvirus protein vIRF1 inhibits STING phosphorylation and activation by preventing TBK1 recruitment to the STING complex [39]. Furthermore, the human cytomegalovirus (HCMV) protein UL82 interferes with the translocation of STING as well as the recruitment of TBK1 and IRF3 [40].

Bovine herpesvirus 1 (BoHV-1) is highly contagious and causes significant economic losses in the cattle industry worldwide [41–43]. BoHV-1, a double-stranded DNA virus with a 135.3 -kb genome, suppresses the host immune response [44]. The BoHV-1 protein bICPo has been shown to degrade IRF3 in a proteasome-dependent manner to suppress IFN-β promoter activity [45–47]. Moreover, the BoHV-1 protein VP8 downregulates the type I IFN pathway early in infection by interacting with STAT1 to prevent nuclear accumulation of STAT1 [48]. Nonetheless, the effect of BoHV-1 infection on the key factor STING in the anti-DNA virus response remains to be elucidated.

In the current study, we identified DDIT3 as a negative regulator of type I IFN signaling induced by the cGAS-STING pathway during BoHV-1 infection. Initially, we found that DDIT3 is able to enhance BoHV-1 replication in MDBK cells. We further uncovered that DDIT3 increases SQSTM1 mRNA and protein levels. Moreover, we observed the interaction between SQSTM1 and STING, and BoHV-1 infection led to SQSTM1-mediated STING degradation via autophagy. Our findings indicate that DDIT3 plays a vital role in innate immune responses against BoHV-1 infection.

## Materials and methods

### Virus and cells

The BoHV-1 strain BarthaNu/67 purchased from the China Veterinary Culture Collection Center was used in all experiments. MDBK cells were infected with BoHV-1 for 1 hour, the supernatant was discarded, and the cells were washed three times using cold phosphate-buffered saline (PBS). The cells were then returned to DMEM with 2% horse serum. The culture medium and cells were harvested at 24 hours post-infection to determine the level of BoHV-1 replication using the Reed-Muench endpoint method.

MDBK cells provided by American Type Culture Collection were maintained in DMEM (88364, Thermo Scientific) supplemented with 10% horse serum (16050122, Gibco) and 1% penicillin-streptomycin. HEK293T cells were cultured in DMEM supplemented with 1% penicillin-streptomycin and 10% fetal bovine serum (16140071, Gibco) at 37°C in 5% CO_2_.

### Cell viability assay

For DDIT3 overexpression and knockdown cell lines, MDBK cells transfected with SQSTM1, ATG5 or scrambled siRNA were seeded into 96-well plates at a density of 2 × 10^3^ cells per well and grown for 12, 24, 48 and 60 h. Cell proliferation and viability were measured by Cell Counting Kit-8.

### Antibodies and reagents

Monoclonal anti-DDIT3 (2895S), anti-Flag (14793S), anti-HA (2367S), anti-p-PI3K (4228S) antibody and anti-LC3A/B (4108S) antibodies were purchased from Cell Signaling Technology (Danvers, MA, USA). Antibodies against total PERK (sc -13073) and cGAS (sc -515777) were obtained from Santa Cruz Biotechnology (Dallas, TX, USA). An anti-p-PERK (ab192591) antibody was obtained from Abcam (Cambridge, UK). An anti-actin rabbit monoclonal antibody (mAb) (AB0033), -GAPDH rabbit mAb (AB0036), -STING rabbit mAb (CY7204), -PI3K rabbit mAb (CY6915) and -SQSTM1 rabbit mAb (CY5546) were obtained from Abways Technology (Shanghai, China). Recombinant bovine IFN alpha (CSB-YP344226BO) was purchased from Cusabio Technology LLC (Cusabio, Houston, TX, USA; https://www.cusabio.com). 3-Methyladenine (3-MA, HY -19312) (MedChemExpress, Monmouth Junction, NJ, USA) and MG132 (HY -13259) (MedChemExpress) were dissolved in dimethyl sulfoxide (DMSO).

### Lentivirus production and establishment of stable overexpressing cell lines

Lentiviral packaging based on the pLVX-IRES-puro vector was used to construct MDBK cell lines stably expressing DDIT3 or SQSTM1. cDNA encoding bovine DDIT3 or SQSTM1 was amplified from MDBK cells and cloned into pLVX-IRES-puro eukaryotic expression vectors using a ClonExpress II One Step Cloning Kit (Vazyme, Nanjing, China). Lentiviruses and stable cell lines were produced according to the product instructions. Briefly, 72 hours after the four lentivirus packaging plasmids were transfected into HEK293T cells, they were passaged no more than 5 times after thawing. The lentiviruses produced were harvested, and MDBK cells were infected with the lentivirus for at least 48 h. Subsequently, the cells were cultured in medium containing puromycin (5 μg/mL), which was replaced every 1–2 days, and puromycin-resistant colonies were selected. Overexpressing cell lines were identified by immunoblotting with anti-DDIT3 or anti-SQSTM1 rabbit monoclonal antibodies. The primers used for amplifying genes are listed in Table 1.

**Table 1.** List of primers used in this study

Name	Sequence (5'-3')
siSQSTM1	GCATTTACATTAAAGAGAA
siATG5	CCTGTATCAGGATGAGATA
DDIT3-F	CCGGAATTCGCCACCatggcagctgagtcactgcct
DDIT3-Flag-R	CGCGGATCCctaCTTGTCATCGTCGTCCTTGTAATCtgcttggtgcagattaacca
SQSTM1-F	CCGGAATTCGCCACCatggcgtcgctcacggtga
SQSTM1-Flag-R	TGCTCTAGActaCTTGTCATCGTCGTCCTTGTAATCcaaaggtggtgggtgttttgaa
qPCR-gC-F	ATGTTAGCGCTCTGGAACC
qPCR-gC-F	CTTTACGGTCGACGACTCC
qPCR-DDIT3-F	GCTGAGTCACTGCCTTTCTCCTTC
qPCR-DDIT3-R	ACACAGGTGCCCCGATTTTCATC
qPCR-IFNβ-F	CCTGTGCCTGATTTCATCATGA
qPCR- IFNβ-R	GCAAGCTGTAGCTCCTGGAAAG
qPCR-MX1-F	AACACCTGACCGCGTACCA
qPCR- MX1-R	GCACGAAGAACTGGATGATCAA
qPCR-ISG15-F	AAGCAGTTCATCGCCCAGAAGATC
qPCR- ISG15-R	CTCTCAGGCCCTGGAGGACAAG
qPCR-IFIT1-F	AGATGGACTGTGAGGAAGGATGGG
qPCR- IFIT1-R	CCTCCAGGCGATAGACAACGATTG

### Establishment of DDIT3 knockdown cell lines

Small interfering RNA sequences (1: 5'-GACTCAAACAGGAAATCGAGC-3,' 2: 5'-GATTGACCGGATGGTTAATCT-3') were inserted into the pYr-Lvsh vector. The generation of lentiviral particles was performed in 293T cells transfected with pYr-Lvsh,pLP1, pLP2 or pLP/VSVG. MDBK cells were infected with lentiviruses as described above for 48 h; 10% horse serum supplemented with puromycin (5 μg/mL) was used to replace the medium containing lentivirus, and fresh medium containing puromycin was changed every 1–2 days until cell colonies were established.

### RNA extraction and real-time PCR analysis

Total cellular RNA was extracted with a Cell Total RNA Isolation Kit (RE -03111, Foregene, Chengdu, China), eluted in 20 μL of water and quantified; reverse transcription was performed with HiScript II Q RT SuperMix for qPCR (R223–01, Vazyme). SYBR Green-based qPCR was performed using TB Green Premix Ex Taq II (TaKaRa, Japan). The relative abundance of transcripts was determined after normalizing the data to the β-actin gene [49]. The related primer sequences are listed in Table 1.

### RNA interference

Small interfering RNA (siRNA) duplexes targeting IFNAR1 and SQSTM1 were obtained from TsingKe Biological Technology (Qingdao, China). siRNA sequences are listed in Table 1. siRNA was transfected into MDBK cells using Lipo3000 and p3000 reagents (Thermo Fisher).

### Protein extraction and western blot analysis

MDBK cells were washed with ice-cold PBS, collected, and then lysed in ice-cold lysis buffer (150 mM NaCl, 5 mM sodium orthovanadate, .1 mM phenylmethylsulfonyl fluoride, 2 mM EDTA, 50 mM Tris-HCl, 1% Triton X-100 and sodium deoxycholate). Approximately 25 µg of total proteins from each cell lysate was subjected to SDS–PAGE. For immunoprecipitation experiments, the extracted proteins were incubated with anti-Flag tag mAb magnetic agarose (MBL, Aichi, Japan) for 1 hour. The beads were washed 4 times using cold PBST, and the immunoprecipitates were resuspended in 5× SDS loading buffer (LT101, EpiZyme Biotechnology, Shanghai, China), boiled for 5 min and resolved by electrophoresis through a 12.5% SDS–PAGE gel (PG113, EpiZyme Biotechnology). The separated proteins were transferred to PVDF membranes and subsequently blocked with 8% nonfat dry milk in TBST (PS103, EpiZyme Biotechnology). The blocked membranes were incubated with the relevant primary antibodies, washed 5 times with TBST, and incubated with appropriate secondary antibodies as previously described [50]. Protein bands were detected using an ECL Chemiluminescence Kit (SQ201, EpiZyme Biotechnology).

### Dual-Luciferase assay

The bovine SQSTM1 promoter region was cloned from bovine genomic DNA and inserted into the pGL3-basic vector to obtain full-length constructs, which were further deleted via PCR to obtain truncated constructs. HEK293T cells cultured on 24-well plates were transfected with luciferase reporter constructs, pRL-TK, and various protein-expressing plasmids. A dual-luciferase reporter assay kit (Promega Corporation, USA) was employed to measure Renilla and firefly luciferase activities following the manufacturer’s instructions.

### Immunofluorescence assays

MDBK cells were seeded on confocal specialized slides, fixed in 4% paraformaldehyde for 15 min, and then permeabilized in cold PBST. Next, MDBK cells were washed with cold PBS 3 times, blocked in 5% fetal bovine serum for 1 h, and incubated with primary antibodies. The cells were incubated with fluorescently labeled secondary antibodies, and then the nuclei were stained using Hoechst stains and finally observed using a Leica SP8 confocal microscope.

### Statistical analyses

Student’s t test was used for statistical significance between two experimental groups. All data are presented as the mean ± standard deviation of three independent experiments, and a P value less than 0.05 was considered to indicate a statistically significant difference. A P value below 0.01 was regarded as a significant difference.

## Results

### BoHV-1 infection upregulates DDIT3 expression

**Figure 1.**
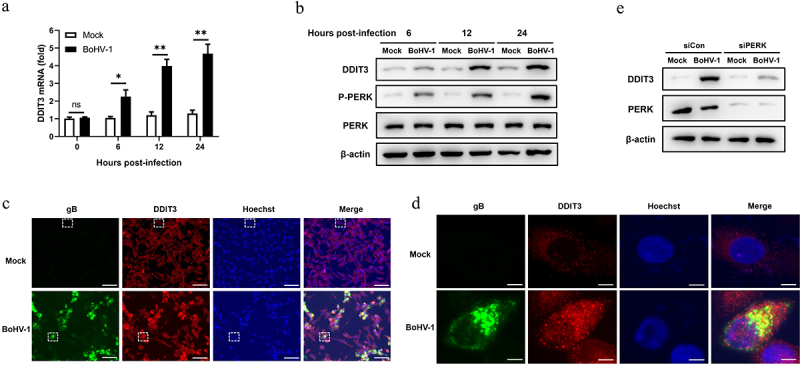
**BoHV-1 infection upregulates the expression of DDIT3**. MDBK cells were infected with BoHV-1 (MOI =0 .1) for the indicated times. (a) Total RNA was harvested to detect the mRNA levels of DDIT3 by qPCR. Means and SD from three independent experiments are shown. **, P < 0.01. (b) DDIT3, PERK, and p-PERK in the total lysates were analyzed by immunoblot analysis at the indicated times. Three replicates were performed for this analysis. (c) MDBK cells were infected with BoHV-1 (MOI = 0.1) for 12 hours. The cells were stained with anti-gB (the BoHV-1 glycoprotein gB) and anti-DDIT3 monoclonal antibodies, and fluorescence images were obtained with confocal microscopy. Bar = 40 μm. (d) Magnified images of the white boxed area in (c). Bar = 5 μm. (e) Thirty-six hours after NC- or DDIT3-overexpressing cells were transfected with siCon or siPERK, the cells were infected with BoHV-1 (MOI =0 .1), and these experiments were conducted three times. Then the cells were harvested at 24 hpi for immunoblot analysis.

Our previous study indicated that BoHV-1 activates the PERK pathway to promote BoHV-1 replication at the post-entry step in the BoHV-1 life cycle [51]. However, the specific mechanism by which the PERK pathway modulates BoHV-1 replication is unresolved. Accordingly, we focused on DDIT3 induced by the PERK pathway to evaluate whether and how it affects BoHV-1 proliferation. To determine the effect of BoHV-1 infection on DDIT3 expression in MDBK cells, the expression between 0 and 24 hours post-infection (hpi) was measured by quantitative real-time PCR and western blotting. Our data showed that BoHV-1 infection upregulated cellular DDIT3 expression (Figure 1(a,b)). We then utilized immunofluorescence assays to examine DDIT3 expression during BoHV-1 infection, and the results confirmed that BoHV-1 infection upregulated DDIT3 expression compared to mock infection (Figure 1(c,d)). PERK inhibition repressed DDIT3 expression in BoHV-1-infected MDBK cells (Figure 1(e)), suggesting that DDIT3 expression is induced by the PERK pathway during BoHV-1 infection. These data clearly demonstrate that BoHV-1 infection triggers the expression of DDIT3, indicating that DDIT3 could assume a potential role in BoHV-1 infection.

### DDIT3 promotes BoHV-1 replication

**Figure 2.**
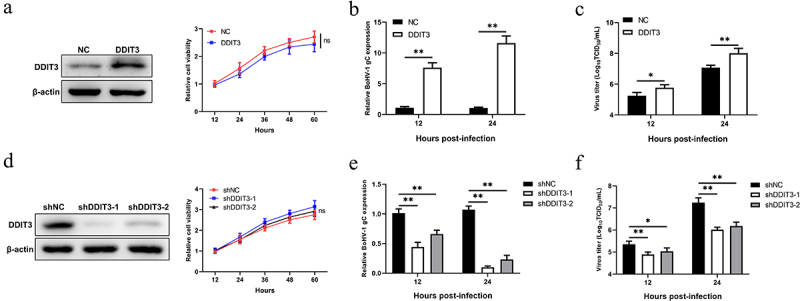
**DDIT3 promotes BoHV-1 replication**. (a) Immunoblot analysis of DDIT3 expression in DDIT3-overexpressing MDBK cells. The CCK-8 assay was used to assess cell viability in DDIT3-overexpressing cell lines. (b,c) DDIT3-overexpressing stable cell lines or negative-control (NC) MDBK cell lines were infected with BoHV-1 (MOI =0 .1) and harvested at 12 and 24 hpi for BoHV-1 gC gene expression and virus titration analyses. Relative levels of BoHV-1 gC mRNA were normalized to β-actin; the fold change was determined by setting the NC group in one of three repeated experiments as 1. (d) Knockdown efficiencies of DDIT3-targeting shrnas in stable MDBK cell lines were assessed by immunoblot analysis. The CCK-8 assay was conducted to determine the cell growth of DDIT3 knockdown cell lines. (e) qPCR analysis of BoHV-1 gC gene expression in stable DDIT3-knockdown cell lines at 12 and 24 hpi. (f) DDIT3-knockdown MDBK cell lines and the corresponding control groups were infected with BoHV-1 (MOI =0 .1) for 12 and 24 hours, and titers of BoHV-1-infected cells were determined.

To explore whether the increase in DDIT3 levels is related to an increased BoHV-1 load, we constructed stable DDIT3-overexpressing stable MDBK cell lines using recombinant lentiviruses. The results of western blotting showed that the overexpression cell lines were successfully constructed, and growth curves measured by CCK-8 assay showed that the growth status of DDIT3-overexpressing cell lines was not significantly different from that of control cell lines (Figure 2(a)). DDIT3 was found to promote BoHV-1 replication, as revealed by measuring relative BoHV-1 glycoprotein C (gC) transcript levels and viral titers at 12 and 24 hpi (Figure 2(b,c)). Next, we further explored the effect of DDIT3 knockdown on BoHV-1 replication; specific small hairpin RNAs (shRNAs) targeting DDIT3 were designed, and lentiviruses were generated to construct cell lines. Knockdown efficiencies were evaluated by western blotting, and cell viability assays for DDIT3 knockdown lines were carried out using the CCK-8 assay (Figure 2(d)). By measuring relative BoHV-1 gC transcript levels and viral titers, DDIT3 knockdown reduced BoHV-1 replication in MDBK cells (Figure 2(e,f)). Collectively, these data suggest that DDIT3 could support BoHV-1 proliferation.

### DDIT3 restrains the antiviral innate immune response during BoHV-1 infection

**Figure 3.**
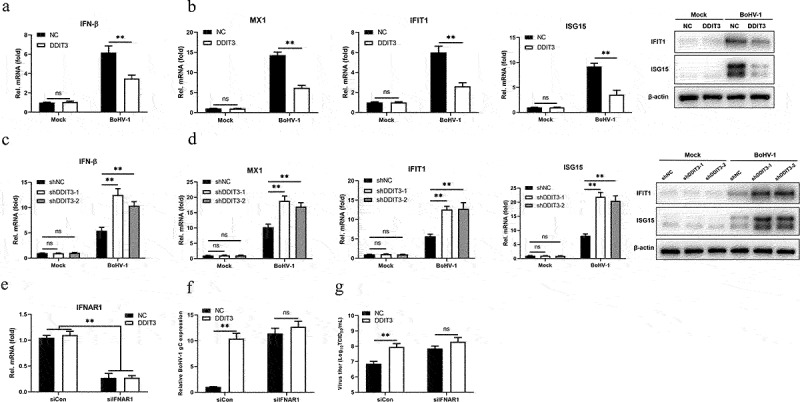
**DDIT3 restrains the antiviral innate immune response during BoHV-1 infection**. (a,b) qPCR analysis of IFN-β, IFIT1, MX1, and ISG15 mRNA levels in DDIT3-overexpressing MDBK cells infected with BoHV-1 (MOI = 0.1) for 12 hours. Immunoblot analysis of ISG15 and IFIT1 expression in DDIT3-overexpressing MDBK cells infected with BoHV-1. (c,d) qPCR analysis of IFN-β, IFIT1, MX1, and ISG15 mRNA levels in DDIT3 knockdown cells infected with BoHV-1 (MOI = 0.1) for 12 hours. Relative IFN-β and ISG mRNA levels were normalized to β-actin; fold change was determined by setting the NC group at 12 hpi in one of three repeated experiments as 1. The means and SD from three independent experiments are shown. **, P < 0.01; ns, not significant. Immunoblot analysis of ISG15 and IFIT1 expression in DDIT3-knockdown MDBK cells infected with BoHV-1. (e) qPCR analysis of the silencing efficiency of IFNAR1 in MDBK cells at 36 hpi. (f and g) After transfection of si-NC or si-IFNAR1 for 36 hours, MDBK cells were infected with BoHV-1 (MOI =0 .1) and harvested at 24 hpi for BoHV-1 gC gene expression and virus titration analyses.

Previous work conducted by our lab and others has demonstrated that the IFN-I pathway plays a critical role in regulating BoHV-1 replication [48,52,53]. Given evidence showing that the UPR induced by flaviviruses regulates early activation of innate antiviral responses [54], DDIT3 is an important effector protein of the UPR, and DDIT3 knockout in T cells could promote antitumor immunity [55]. Next, we sought to determine whether BoHV-1 infection is capable of DDIT3 regulating innate antiviral responses. The results showed that DDIT3 reduced the mRNA levels of BoHV-1-induced IFN-β, MX1, IFIT1 and ISG15, as well as the protein levels of IFIT1 and ISG15 (Figure 3(a,b)). Next, we examined the mRNA levels of IFN-β and ISGs in DDIT3 knockdown cell lines. As shown in Figure 3(c,d), DDIT3 knockdown promoted the mRNA levels of IFN-β, MX1, IFIT1 and ISG15, as well as the protein levels of IFIT1 and ISG15.

To determine whether DDIT3 facilitates BoHV-1 replication primarily by inhibiting the type I IFN response, the effect of DDIT3 on BoHV-1 proliferation was measured in type I IFN-deficient MDBK cells. We used specific siRNA targeting IFNAR1 to block the type I IFN response and confirmed IFNAR1 mRNA levels in DDIT3-overexpressing MDBK cells by qPCR (Figure 3(e)). The effect of DDIT3 on BoHV-1 replication was examined by measuring gC gene mRNA levels and viral titers in IFNAR1-deficient cells. According to the results, there was no significant difference in BoHV-1 gC transcript levels (Figure 3(F)) or the BoHV-1 viral titer (Figure 3(g)), indicating that DDIT3 promotes BoHV-1 replication mainly by blocking the IFN antiviral response. These results collectively indicate that DDIT3 suppresses the antiviral innate immune response during BoHV-1 infection.

### DDIT3 targets STING to inhibit the IFN antiviral response

**Figure 4.**
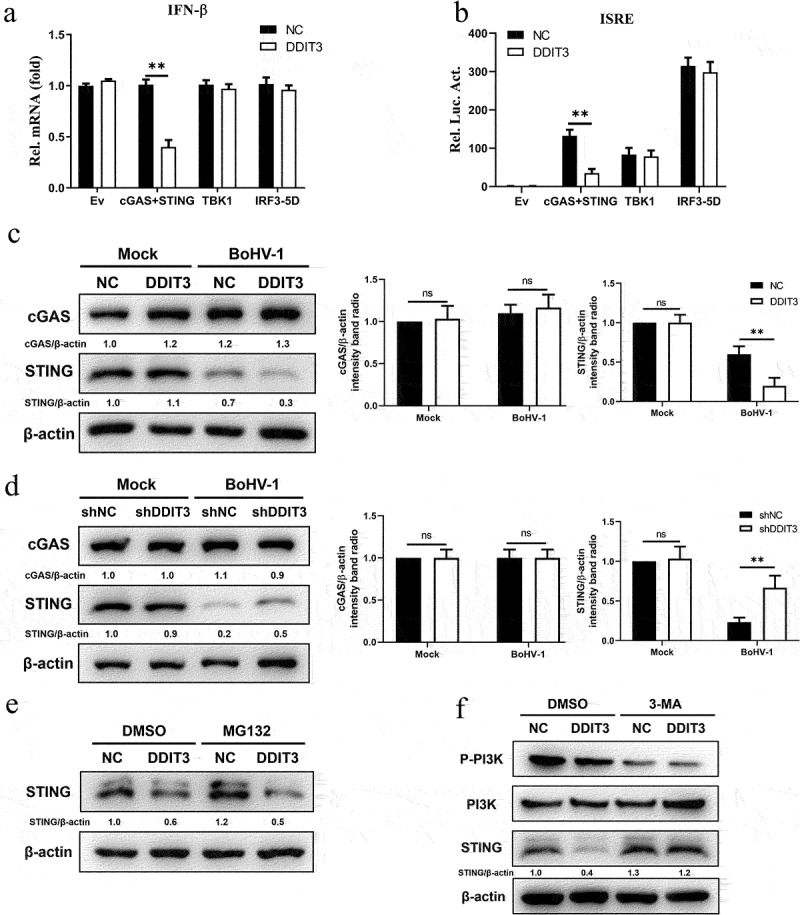
**DDIT3 targets STING to inhibit the innate immune response**. (a) IFN-β mRNA levels in cells transfected with expression plasmids for cGAS+ STING, TBK1 or IRF3-5D along with the control or DDIT3 vector for 24 hours. (b) Luciferase (Luc) activity of the IFN-β promoter reporter in HEK293 cells co-transfected with expression plasmids for cGAS+ STING, TBK1 or IRF3-5D together with the control or DDIT3 vector. (c) Immunoblot analysis of cGAS and STING protein levels in negative-control (NC) and DDIT3-overexpressing MDBK cells infected with BoHV-1 (MOI =0 .1). Relative densitometry of each band was performed by measuring band intensity with AlphaView software version 3.4. Graphs on the right show quantification of western blot data from three independent experiments. (d) Immunoblot analysis of cGAS and STING protein levels in DDIT3-knockdown or shNC-transfected MDBK cells infected with BoHV-1 (MOI =0 .1). Densitometry of each band was performed by measuring band intensity with AlphaView software. Experiments were repeated three times. (e, f) Immunoblot analysis of STING levels in DDIT3-overexpressing MDBK cells pretreated with MG132 (0.5 mM) or 3-MA (2 mM) followed by infection with BoHV-1 (MOI = 0.1) for 24 hours. the western blot experiment was repeated independently three times with similar results.

To identify the target component of DDIT3 in the IFN antiviral response during BoHV-1 infection, the effects of DDIT3 on the IFN-β transcript levels mediated by key components of the cGAS–STING pathway were measured. The results showed that DDIT3 suppressed the transcription of IFN-β induced by overexpression of cGAS+ STING but not TBK1- or IRF3-5D-induced (Figure 4(a)). Similarly, ISRE promoter activity induced by cGAS+ STING was decreased in HEK293 cells transfected with a DDIT3 expression plasmid, whereas TBK1- and IRF3-5D-induced IFN-β promoter activity was not (Figure 4(b)). These results show that DDIT3 blocks transmission of the cGAS–STING pathway by inhibiting cGAS or STING. Therefore, we determined cGAS and STING protein levels by western blotting and observed that DDIT3 downregulated STING expression but did not affect cGAS protein levels (Figure 4(c)). Consistently, DDIT3 knockdown upregulated STING protein levels but had no effect on cGAS expression (Figure 4(d)). Next, we examined whether the degradation of STING caused by DDIT3 occurs through the proteasome or autophagolysosome pathway according to our previous study [56]. Downregulation of STING protein abundance by DDIT3 overexpression was rescued by treatment with the 3-methyladenine (3-MA) autophagy inhibitor but not with the proteasome inhibitor MG132 (Figure 4(e,f)). Taken together, our findings reveal that DDIT3 promotes the degradation of STING via autophagic degradation during BoHV-1 infection.

### SQSTM1 promotes BoHV-1 replication by inhibiting IFN antiviral responses

**Figure 5.**
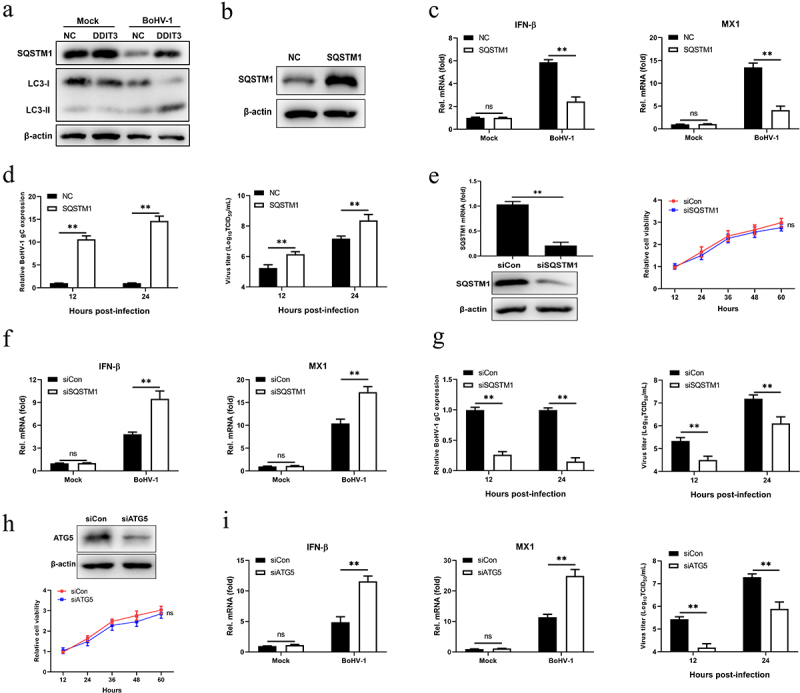
**SQSTM1 promotes BoHV-1 replication by inhibiting IFN antiviral responses**. (a) Immunoblot analysis of the indicated protein in NC- and DDIT3-overexpressing MDBK cells infected with BoHV-1 (MOI = 0.1). Three replicates were used for immunoblot analysis. (b) Immunoblot analysis of the SQSTM1 protein in SQSTM1-overexpressing or empty retrovirus-transduced MDBK cells. (c) qPCR analysis of IFN-β and MX1 mRNA levels in DDIT3-overexpressing or control cells infected with BoHV-1 (MOI = 0.1). (d) DDIT3-overexpressing cells were infected with BoHV-1 (MOI = 0.1) and harvested for viral gC gene expression analyses and virus titration analyses. (e) The silencing efficiency of siRNA targeting SQSTM1 in MDBK cells was measured by qPCR and immunoblotting. CCK-8 assays were used to measure the viability of cells after siRNA transfection. (f) qPCR analysis of IFN-β and MX1 mRNA levels in DDIT3-knockdown cells infected with BoHV-1 (MOI =0 .1). (g) DDIT3-knockdown cells were infected with BoHV-1 and harvested at 12 and 24 hpi for gC gene expression and virus titration analyses. (h) The knockdown efficiency of siRNA targeting ATG5 in MDBK cells was assessed by immunoblotting. The viability of MDBK cells after MDBK transfection with siRNA was assessed by CCK-8 assays. (i) At 36 h after siATG5 transfection, MDBK cells infected with BoHV-1 (MOI = 0.1) were harvested for qPCR and virus titration. The means and SD from three independent experiments are shown. **, P <0 .01; ns, not significant.

Previous studies have demonstrated that STING degradation occurs in the lysosomal compartment and is mediated by SQSTM1-dependent autophagy [57,58]. Given the essential function of SQSTM1 in DNA-stimulated STING degradation, we analyzed SQSTM1 in BoHV-1-infected DDIT3-overexpressing cell lines and found that SQSTM1 protein levels were upregulated and the conversion of LC3-I to LC3-II was enhanced (Figure 5(a)). We surmised that DDIT3 could promote autophagic degradation of STING by upregulating the expression of SQSTM1 in BoHV1-infected MDBK cells. Thus, to explore whether SQSTM1 inhibits IFN antiviral responses, we generated SQSTM1-overexpressing MDBK cells using recombinant lentiviruses and performed western blotting (Figure 5(b)). SQSTM1 inhibited the expression of IFN-β and MX1, indicating that it could suppress the type I IFN response in MDBK cells (Figure 5(c)). Moreover, the expression of the BoHV-1 gC gene and the BoHV-1 titer were significantly increased in SQSTM1-overexpressing cells during viral infection (Figure 5(d)). To confirm the negative effect of SQSTM1 on IFN antiviral responses, we utilized siRNAs to knockdown SQSTM1 and then analyzed IFN production and BoHV-1 virus titers. Knockdown efficiencies of siRNAs targeting SQSTM1 were assessed in MDBK cell lines by qPCR and western blotting; the CCK-8 assay was used to measure MDBK cell viability in response to transfection with SQSTM1 siRNA (Figure 5(e)). IFN-β and MX1 expression was significantly increased in SQSTM1-knockdown cells, and SQSTM1 knockdown significantly reduced BoHV-1 replication in MDBK cells (Figure 5(F,g)). ATG5 is a known effector of STING-induced autophagy induction [59]. Likewise, ATG5 knockdown inhibited BoHV-1 replication in MDBK cells by promoting IFN antiviral responses (Figure 5(h,i)). These data indicate that SQSTM1 supports the proliferation of BoHV-1 cells by inhibiting IFN antiviral responses.

### SQSTM1 inhibits innate immunity by promoting STING degradation in MDBK cells

**Figure 6.**
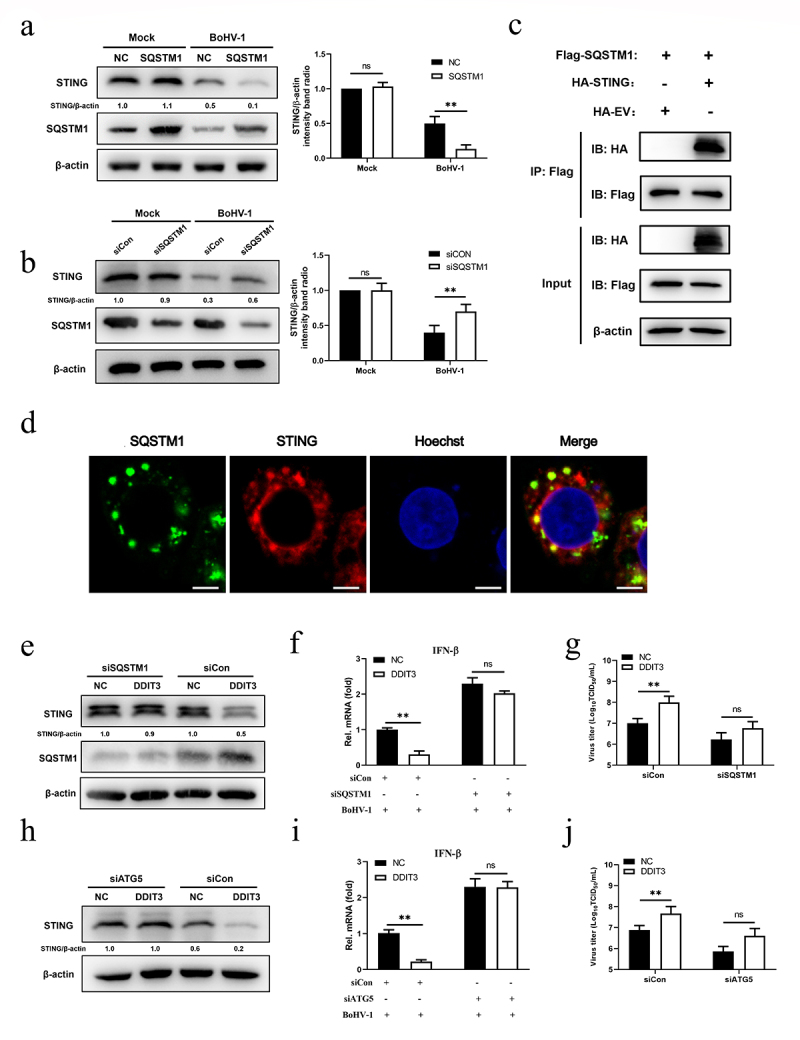
**SQSTM1 inhibits innate immunity by promoting STING degradation in MDBK cells**. (a) Immunoblot analysis of SQSTM1 and STING protein levels in NC- and DDIT3-overexpressing MDBK cells infected with BoHV-1 (MOI = 0.1), experiments were conducted three times. (b) At 48 hours post-transfection with si-Con or si-SQSTM1, MDBK cells were infected with BoHV-1 (MOI = 0.1) and harvested at 24 hpi for immunoblot analysis. Graphs on the right show quantification of western blot data from three independent experiments. Relative intensity was quantified and analyzed based on densitometry using AlphaView software. (c) Coimmunoprecipitation and immunoblot analysis of extracts of 293T cells transfected with Flag-SQSTM1 and HA-EV or HA-STING. (d) Confocal microscopy analysis of colocalization of bovine SQSTM1 and bovine STING. HEK 293T cells transfected with Flag-SQSTM1 and HA-STING were subjected to immunofluorescence analysis using Flag- and HA-specific antibodies. Bar = 5 μm. (e, f, and g) Thirty-six hours after NC- or DDIT3-overexpressing cells were transfected with si-Con or si-SQSTM1, the cells were infected with BoHV-1 (MOI =0 .1) and harvested at 24 hpi for immunoblot analysis (e), qPCR (f) or virus titration (g). (h, i, and j) At 36 h after siATG5 transfection, DDIT3-overexpressing and control cells were infected with BoHV-1 (MOI = 0.1), and the cells were harvested at 24 hpi for immunoblot analysis (h), qPCR (i) or virus titration (j). For E and H, the western blot experiment was repeated independently three times with similar results. The means and SD from three independent experiments are shown. **, P < 0.01; ns, not significant.

After DNA stimulation, SQSTM1 strongly localizes to the same region as STING in human acute monocytic leukemia cells and then mediates autophagic degradation of STING [57]. Hence, we examined whether SQSTM1 induced by DDIT3 suppresses innate immunity against BoHV-1 infection by negatively modulating the protein levels of STING. SQSTM1 reduced STING protein levels in BoHV-1-infected MDBK cells at 12 and 24 hpi (Figure 6(a)), whereas SQSTM1 knockdown upregulated the protein level of STING at 12 and 24 hpi (Figure 6(b)). Next, we confirmed the interaction between SQSTM1 and STING in HEK293T cells by co-IP assays and confocal immunofluorescence assays (Figure 6(c,d)). Notably, the STING protein level was restored, IFN-β transcription inhibition was ameliorated, and viral titers were reduced when the expression of SQSTM1 was knocked down with siRNA in DDIT3-overexpressing cell lines during BoHV-1 infection (Figure 6(e–g)). Moreover, STING degradation and IFN-β inhibition were also ameliorated, and viral titers were reduced when ATG5 expression was knocked down in DDIT3-overexpressing cell lines (Figure 6(h–j)). Together, these data indicate that SQSTM1 inhibits innate immunity against BoHV-1 infection by promoting the degradation of STING in MDBK cells.

### The increase in SQSTM1 expression is DDIT3 dependent in MDBK cells

**Figure 7.**
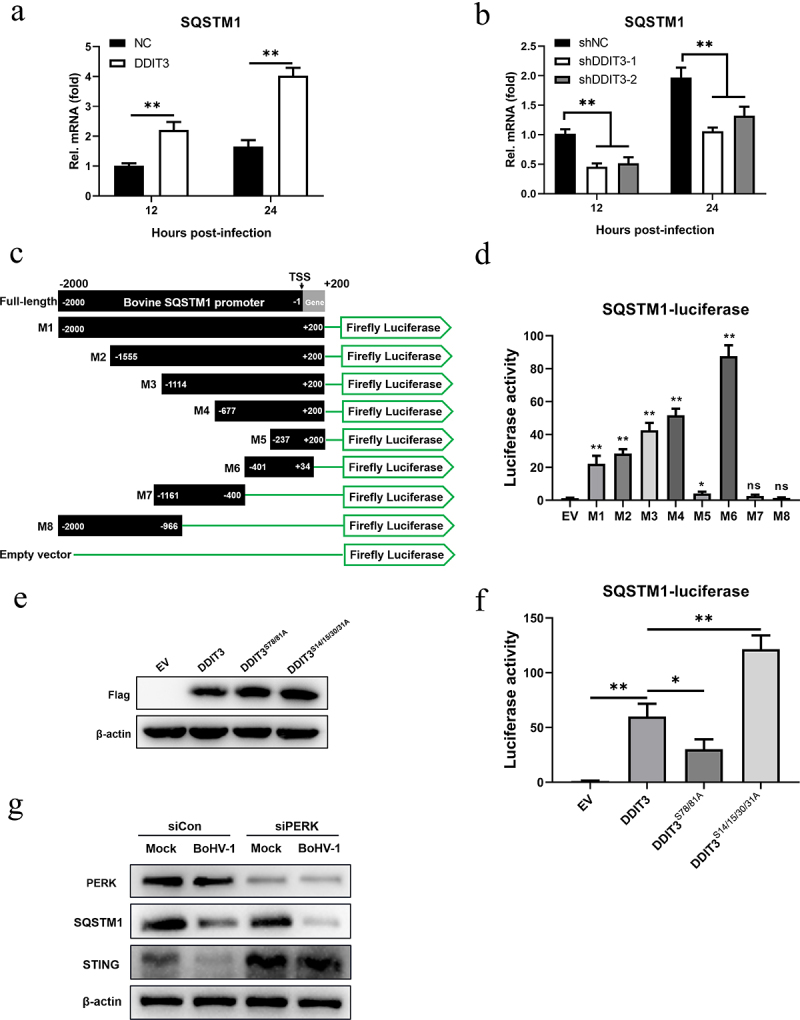
**The increase in SQSTM1 expression is DDIT3 dependent in MDBK cells**. (a) qPCR analysis of SQSTM1 mRNA levels in DDIT3-overexpressing or control cells infected with BoHV-1 (MOI =0 .1) at 12 and 24 hpi. (b) qPCR analysis of SQSTM1 mRNA levels in DDIT3 knockdown cells at 12 and 24 h after BoHV-1 infection (MOI =0 .1). (c, d) HEK293T cells were co-transfected with a series of truncated SQSTM1 promoter constructs (M1 to M8) (c) together with a Renilla luciferase reporter vector and analyzed for dual luciferase activity (d). (e) Immunoblot analysis of bovine DDIT3 and mutants in HEK293T cells. (f) Expression of luciferase (Luc) activity of the SQSTM1 promoter (M6) reporter in HEK293 cells co-transfected with DDIT3 or mutants together with the pRL-TK-luc vector. The means and SD from three independent experiments are shown. **, P <0 .01; *, P < 0.05; ns, not significant. (g) At 36 h after siPERK transfection, MDBK cells were infected with BoHV-1 (MOI =0 .1), and the cells were harvested at 24 hpi for immunoblot analysis.

Next, we sought to elucidate the mechanism by which DDIT3 affects SQSTM1 expression during BoHV-1 infection. We hypothesized that DDIT3 acts as a transcription factor to promote the expression of SQSTM1 at the mRNA level in MDBK cells. Therefore, we measured the effects of SQSTM1 mRNA levels in DDIT3-overexpressing or DDIT3-silenced cells during BoHV-1 infection. In DDIT3-overexpressing cells, the mRNA levels of SQSTM1 were increased by qPCR (Figure 7(a)), and compared with control cells, SQSTM1 mRNA levels were significantly reduced after DDIT3 silencing (Figure 7(b)). To determine the boundaries of the bovine SQSTM1 promoter, we amplified 2000 bp of the SQSTM1 promoter, constructed different truncated promoters (designated M1-M8) (Figure 7(c)) and assessed the ability of these truncated promoters to direct luciferase expression in HEK293T cells (Figure 7(d)). As the truncated M6 promoter showed the highest expression activity, the SQSTM1 core promoter spanned from positions −401 to +34.

It was previously reported that phosphorylation of serine residues 78 and 81 of DDIT3 enhances its transcriptional activity but that phosphorylation of serine residues 14/15/30/31 inhibits its transcriptional activity [60,61]. Therefore, we constructed mutants of bovine-derived DDIT3 with mutations in two (both S78A and S81A) or four (S14A, S15A, S30A, and S31A) conserved sites and evaluated expression by western blotting (Figure 7(e)). Next, we examined whether DDIT3 and 2 mutants were able to affect the luciferase activity of the bovine SQSTM1 promoter M6. Compared with the empty vector, DDIT3 significantly increased luciferase expression from M6 in HEK293T cells (Figure 7(F)). We also found that mutant DDIT3 (S14/15/30/31A) activated luciferase expression of the bovine SQSTM1 promoter more than 2-fold compared with its expression in cells transfected with wild-type DDIT3 (Figure 7(F)). Furthermore, mutant DDIT3 (S78/81A) exhibited significantly inhibited luciferase expression of the SQSTM1 promoter compared with that of normal DDIT3, indicating that the transcriptional activity of DDIT3 is critical for the transcription of SQSTM1 (Figure 7(F)). Collectively, these results suggest that the increase in SQSTM1 expression is DDIT3 dependent in MDBK cells.

We previously found that BoHV-1 activates the PERK pathway to promote BoHV-1 replication [51]. Here, upregulation of DDIT3 expression induced by BoHV-1 infection via the PERK pathway was confirmed (Figure 1(b,e)). Next, we examined the expression of STING and SQSTM1 in BoHV-1-infected MDBK cells using siRNA to silence the expression of PERK. The results showed that the degradation of STING caused by BoHV1 infection was rescued and SQSTM1 protein levels were reduced after PERK silencing. These data indicate that the PERK pathway induced by BoHV-1 may inhibit innate immunity through the DDIT3-SQSTM1-STING pathway, which ultimately promotes viral replication.

## Discussion

DDIT3, a downstream gene target of endoplasmic reticulum stress, affects the replication of many viruses. We recently reported that DDIT3 targets MAVS to inhibit innate immunity, thereby promoting the replication of BVDV (an RNA virus). The main pathways that induce host cells to produce type I interferon (IFN-I) after RNA or DNA virus infection differ. Host cells usually activate the cGAS-STING pathway to induce IFN-I production to fight DNA virus infection, yet whether DDIT3 regulates innate immunity to participate in the replication of DNA viruses remains unclear. Here, we found that DDIT3 negatively regulated the cGAS-STING pathway by targeting STING in MDBK cells. DDIT3 promotes STING degradation through the SQSTM1-mediated autophagy-lysosome pathway, and upregulation of SQSTM1 expression was found to be DDIT3 dependent. This is the first report that DDIT3 suppresses innate immunity to regulate DNA virus replication.

After viruses invade cells, viral proteins are synthesized in the ER, which usually leads to protein accumulation and the UPR to restore ER homeostasis. Our previous study showed that BoHV-1 activates all three UPR pathways in MDBK cells. And BoHV-1 activates the PERK pathways to promote its replication [51]. Several studies have reported that DDIT3, a downstream gene target of the PERK pathway activated by the UPR, affects the replication of some viruses. The HCV core protein activates the PERK pathway to enhance the expression of DDIT3, promoting HCV replication [62,63]. The host protein Irgm3 strongly inhibits the activation of ER stress responses, which further attenuates the induction of DDIT3 and reduces coxsackievirus B3 (CVB3) replication [64]. Moreover, the PERK-eIF2alpha-DDIT3 pathway supports replication of Newcastle disease virus (NDV) by promoting apoptosis and inflammation [65]. Nonetheless, it remains unclear whether DDIT3 is also involved in the BoHV-1 life cycle.

In this study, we found that DDIT3 was upregulated in BoHV-1-infected MDBK, suggesting that DDIT3 plays a potential role in viral replication (Figure 1). gC gene expression and BoHV-1 titers were significantly increased at 12 and 24 hpi in DDIT3-overexpressing MDBK cells; in DDIT3-knockdown cells, the virus titer was decreased by approximately 8-fold at 24 hpi compared with the control group (Figure 2). It has been reported that foot-and-mouth disease virus (FMDV) can activate the PERK pathway and mediate autophagy to inhibit the antiviral IFN response[66]. Considering the important role of the IFN-I pathway in regulating BoHV-1 replication, we examined the innate antiviral responses of DDIT3-overexpressing or DDIT3-knockdown cell lines infected with BoHV-1 and found that DDIT3 inhibited IFN-β and ISG expression in BoHV1-infected MDBK cells (Figure 3). VSV infection could inhibit IFN-I production and antiviral defenses via a PERK-dependent pathway [67]. HCV-mediated activation of the UPR-autophagy pathways could inhibit IFN-β production [62]. Here, we demonstrate that DDIT3 promotes DNA virus replication by inhibiting IFN-I responses, indicating that induction of the PERK pathway of the UPR by BoHV-1 infection can limit IFN-I production.

According to our recent report, DDIT3 induced by BVDV (RNA virus) infection inhibits IFN-I production by degrading MAVS. However, DDIT3 does not degrade MAVS in the presence of BoHV-1 (DNA virus) infection, indicating that DDIT3 inhibits IFN-I in different ways during infection by DNA and RNA viruses. We found that STING was a candidate gene targeted by DDIT3 in the cGAS-STING pathway (Figure 4). The STING protein plays an important role in the anti-DNA virus response, and many viruses have evolved strategies to disrupt STING to inhibit the cGAS-STING pathway. For example, human cytomegalovirus (HCMV) protein IE86 facilitates proteasome-dependent degradation of STING [68], and the HCMV protein UL94 impairs STING dimerization and translocation to interfere with the interaction between STING and TBK1 [69]. The Marek’s disease virus (MDV) oncoprotein Meq blocks recruitment of TBK1 and IRF7 to the STING complex and inhibits the antiviral type I IFN response [70]. Tripartite motif-containing 7 (TRIM7/RNF90/GNIP), which is induced by HSV-1 infection, promotes K48-linked ubiquitination of STING to cause degradation of STING [71]. In addition, PTPN1/2 downregulates STING expression via the ubiquitin-independent 20S proteasome [72]. In the current study, overexpression of DDIT3 did not regulate TRIM7 or PTPN1/2 protein expression to promote STING degradation (data not shown). Therefore, we evaluated whether STING degradation occurs in BoHV-1-infected DDIT3-overexpressing cells through the autophagy-lysosomal or ubiquitin-proteasome degradation pathways. Our data revealed that the rescue of STING protein degradation only occurred in DDIT3-overexpressing MDBK cells treated with an autophagy inhibitor (3-MA); as a proteasome inhibitor (MG132) did not restore the protein level of STING, DDIT3 appeared to promote STING autophagic pathway-dependent degradation during BoHV-1 infection (Figure 4). To date, there are no reports of DDIT3 targeting STING to affect the innate immune response during BoHV-1 infection. Previous studies have shown that late-stage STING vesicles are sorted to lysosomes for trafficking-mediated STING degradation [73] and that the autophagic cargo receptor SQSTM1 mediates the autophagic degradation of STING in human acute monocytic leukemia cells under HSV-1 stimulation [57]. Accordingly, we first verified that BoHV-1 infection induces SQSTM1-mediated autophagy and that DDIT3 upregulates the expression of SQSTM1 (Figure 5(a)). Further analysis revealed that SQSTM1 promotes BoHV-1 replication by inhibiting IFN antiviral responses, which indicates that SQSTM1 also plays a key role in regulating the cGAS-STING pathway in MDBK cells (Figure 5). We then found that SQSTM1 promotes the degradation of STING in cells infected with BoHV-1. Notably, SQSTM1 knockdown in DDIT3-overexpressing cell lines restored STING protein levels and IFN-β inhibition (Figure 6). In addition, the transcriptional activity of DDIT3 determines SQSTM1 expression in MDBK cells (Figure 7). These data suggest that DDIT3 could promote the degradation of STING in MDBK cells through upregulation of SQSTM1 expression. SQSTM1 has been reported to be a substrate of autophagic degradation [74]. Dengue virus-mediated induction of the UPR is required for autophagy activation [75]. Here, we found that DDIT3, a critical effector of the UPR, enhances autophagy activation to inhibit IFN-I responses during BoHV-1 infection, indicating that the UPR may link autophagy to innate immunity via DDIT3.

**Figure 8.**
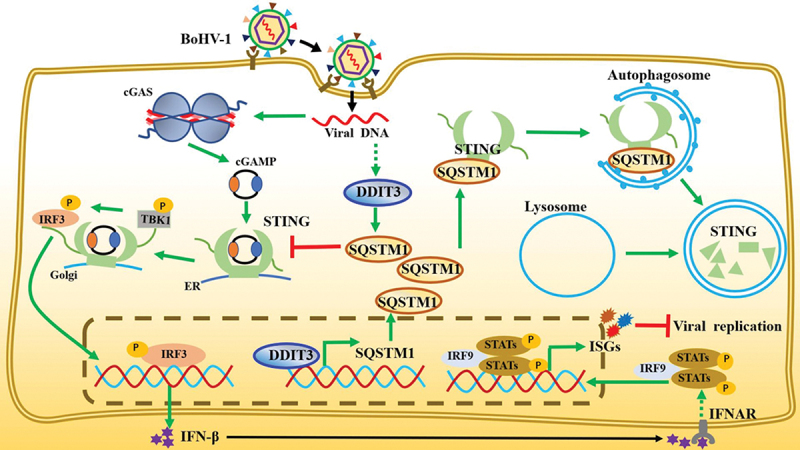
**Model depicting the mechanism by which DDIT3-SQSTM1-STING pathway activation promotes BoHV-1 infection**. BoHV-1 infection-induced upregulation of DDIT3 expression increases SQSTM1 expression by transcriptional activity. SQSTM1 promotes the autophagic degradation of STING, thereby blocking signal transmission by the cGAS-STING pathway and inhibiting the expression of IFN-I and ISGs.

In summary, our study proposes a new model in which upregulation of DDIT3 gene expression caused by BoHV-1 infection promotes STING protein degradation, thereby antagonizing the cGAS-STING pathway and promoting virus replication (Figure 8). Our data demonstrate that DDIT3 acts as a transcription factor for SQSTM1 to promote its expression, which results in STING degradation via the SQSTM1‐dependent autophagy pathway, inhibiting the expression of IFN-Is and ISGs and ultimately terminating the transmission of innate immune responses against DNA viruses. Thus, our work reveals novel roles for DDIT3 in inhibiting the cGAS-STING pathway and in facilitating DNA virus infection.

## Data Availability

The data that support the findings of this study are available from the corresponding author Hongmei Wang or Honbin He.
